# A Compact V-Band Temperature Compensation Low-Noise Amplifier in a 130 nm SiGe BiCMOS Process

**DOI:** 10.3390/mi15101248

**Published:** 2024-10-11

**Authors:** Yi Shen, Jiang Luo, Wei Zhao, Jun-Yan Dai, Qiang Cheng

**Affiliations:** 1 School of Electronics and Information, Hangzhou Dianzi University, Hangzhou 310018, China; 2 State Key Laboratory of Millimeter Waves, Southeast University, Nanjing 210096, China

**Keywords:** millimeter-wave (mm-wave), V-band, SiGe BiCMOS, temperature compensation, low-noise amplifier

## Abstract

This paper presents a compact V-band low-noise amplifier (LNA) featuring temperature compensation, implemented in a 130 nm SiGe BiCMOS process. A negative temperature coefficient bias circuit generates an adaptive current for temperature compensation, enhancing the LNA’s temperature robustness. A T-type inductive network is employed to establish two dominant poles at different frequencies, significantly broadening the amplifier’s bandwidth. Over the wide temperature range of −55 °C to 85 °C, the LNA prototype exhibits a gain variation of less than 1.5 dB at test frequencies from 40 GHz to 65 GHz, corresponding to a temperature coefficient of 0.01 dB/°C. At −55 °C, 25 °C, and 85 °C, the measured peak gains are 25.5 dB, 25 dB, and 24.4 dB, respectively, with minimum noise figures (NF) of 3.0 dB, 3.5 dB, and 4.2 dB, and DC power consumptions of 22.3 mW, 27.6 mW, and 34.4 mW. Moreover, the total silicon area of the LNA chip is 0.37 mm^2^, including all test pads, while the core area is only 0.09 mm^2^.

## 1. Introduction

The V-band spans a wide frequency spectrum from 40 GHz to 75 GHz, making it an ideal candidate for short-range communications and satellite detection due to its short wavelength and sensitivity to atmospheric conditions [1]. These applications have driven the increasing demand for V-band front-end modules, particularly in the 60 GHz band, which has been standardized by IEEE 802.15.3c [2,3]. Given the complex environments encountered in satellite detection and communications, minimizing receiver performance fluctuations across varying ambient temperatures is crucial to ensuring stable and reliable millimeter-wave (mm-wave) front ends. As the first active component in a millimeter-wave receiver, the low-noise amplifier (LNA) amplifies weak signals received by the antenna while suppressing noise in subsequent stages, ensuring a good signal-to-noise ratio. Consequently, high-performance LNAs with robust temperature tolerance have garnered significant attention.

III-V compound processes, such as indium phosphide (InP) and gallium arsenide(GaAs), are known for their high electron mobility, low-noise figure (NF), and high-power density, making them perfect for mm-wave applications, especially in the design of LNAs and power amplifiers (PAs), where they offer exceptional performance. For example, a 50–75 GHz broadband LNA using the 35 nm InP HEMT process was developed, providing a gain of 15–25 dB and an optimal NF of up to 2.2 dB [4]. Using self-biasing and negative feedback techniques, Macro et al. implemented two broadband LNAs in the V-band and Q-band with a 70 nm GaAs process [5,6]. The V-band LNA achieved an NF of less than 1.7 dB and a gain of 15 dB, while the Q-band LNA achieved an NF of less than 1.55 dB and a peak gain of 19 dB. While compound-based LNAs offer superior performance, they generally come with higher power consumption and manufacturing costs, and they lag behind CMOS/SiGe BiCMOS processes in terms of integration and compatibility [7].

Due to aggressive device scaling, CMOS/SiGe BiCMOS technology has become a strong competitor to III-V group semiconductors for high-performance, cost-effective mm-wave integrated circuits. Silicon-based LNAs in V-band are published in [8,9,10,11,12,13,14], with most of the literature providing measured results at room temperature (25 °C) [8,9,10,11], leaving their temperature tolerance unknown. Xu et al. implemented a 60 GHz wideband LNA using an advanced 22 nm FD-SOI process. Benefiting from gain distribution techniques, this LNA achieved a 3 dB bandwidth of 18.5 GHz and a peak gain of 20.2 dB, with a minimum noise figure (NF) of 3.3 dB and power consumption of 8.1 mW. Through innovations in topology and circuit bias point design, the work in [9] reported an ultra-low-power wideband CMOS LNA in the V-band, consuming only 8.6 mW at 60 GHz with a low supply voltage of 0.8 V. It achieved a peak gain of 20.4 dB, though the NF of 5 dB leaves room for improvement. Leveraging digitally controlled current reuse, Chang et al. [10] developed a low-power CMOS digitally controlled variable-gain LNA, achieving a gain control range of 6.6–19.8 dB with a 3 dB bandwidth of over 10 GHz. However, while optimizing for low power and wideband performance, compromises were made in NF and input return loss. References [12,13,14] propose LNAs with temperature tolerance characteristics. By utilizing a bias circuit based on current mirrors and operational amplifiers (OPAMPs), these designs effectively mitigate the gain reduction of the LNA caused by rising temperatures. The prototype of the 60 GHz LNA shows a gain variation of less than 1.7 dB over the temperature range of −20 °C to 100 °C, with a 3 dB bandwidth exceeding 11.5 GHz, although the peak gain is only 9.9 dB. Reference [13] describes a G-band SiGe BiCMOS LNA that employs a bias network based on positive temperature coefficient (PTAT) and negative temperature coefficient (CTAT) resistors. This prototype exhibits a gain variation of less than 2.2 dB in the frequency range of 180–210 GHz over temperatures from −20 °C to 80 °C, with a total power consumption of 35 mW. A standardized temperature compensation design method for mm-wave amplifier design is presented in reference [14], which adjusts the bias circuit by optimizing the reference voltage. This method has been validated in a Ka-band amplifier, although the temperature compensation bias circuit is relatively complex and occupies a significant area.

In this paper, a SiGe BiCMOS V-band wideband LNA with excellent temperature tolerance is proposed, featuring high gain, low noise, and a compact core area. The design employs a current mirror-based adaptive temperature compensation circuit that is simple, easy to implement, and has a small footprint, effectively counteracting temperature-induced variations in gain and NF. Furthermore, a T-type pole-tuning network significantly extends the LNA’s bandwidth. The paper is organized as follows: Section 2 details the circuit design of the proposed LNA; Section 3 presents experimental results to verify the design methodology; and the conclusions are summarized in Section 4.

## 2. Circuit Analysis and Design

**Figure 1:**
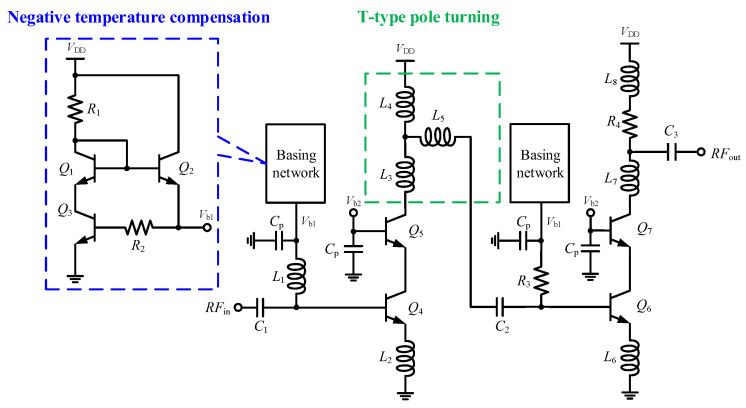
Schematic of the proposed LNA.

Figure 1 illustrates the circuit diagram of the proposed V-band temperature-compensated LNA, which consists of two cascaded common emitter–common base (CE-CB) amplifier stages, a T-type pole tuning network, and a temperature-compensated bias circuit. The CE-CB topology was chosen over common emitter (CE) and common base (CB) configurations because it offers higher voltage gain, better reverse isolation, and the ability to simultaneously optimize noise figure and power matching. The T-type pole tuning network, made up of inductors *L*_3_, *L*_4_, and *L*_5_, is used for interstage matching in the LNA, allowing for adjustment of the high- and low-frequency dominant poles to achieve a flat wideband gain–frequency response. To counteract performance degradation due to temperature variations, a negative temperature coefficient bias circuit generates an adaptive bias current for the base terminals of heterojunction bipolar transistors (HBTs) *Q*_4_ and *Q*_6_, significantly improving the LNA’s temperature robustness.

### 2.1. Size of Transistor and Bias Conditions

It is crucial to determine the sizes and biasing conditions of the active devices before designing the passive components, which ultimately influence the overall performance, such as the minimum noise figure (NF_min_), maximum gain (*G*_max_), and input 1 dB compression point (IP_1dB_). For LNA designs based on current-restricted HBTs, the current density (*J*_C_) comprehensively reflects the size and biasing state. According to [15], at the same frequency, transistors of different sizes can achieve NF_min_ or *G*_max_ at the same current density. *J*_C_ of HBTs can be expressed by emitter current per emitter length, written as follows:

(1) $$J_{C}=\frac{I_{C}}{L_{E}}$$

**Figure 2:**
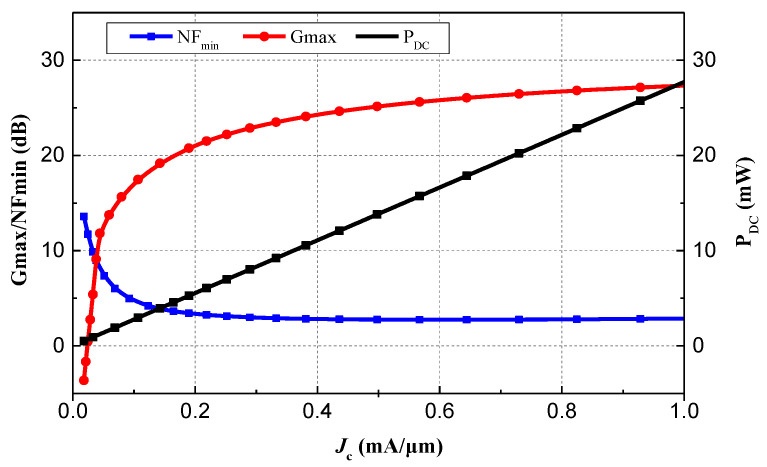
Simulated *G*_max_, NF_min_, and *P*_DC_ with variations of *J*_C_ at 60 GHz and 25 °C when *L*_E_ = 8 μm.

The emitter width of HBTs is typically selected to be a minimum of 120 nm for optimal high-frequency performance. Noting that in addition to *G*_max_ and NF_min_, the power consumed (*P*_DC_) should also be considered. The variation of *G*_max_, NF_min_, and *P*_DC_ verse current density when *L*_E_ = 8 um at a frequency of 60 GHz is shown in Figure 2. To comprehensively evaluate the effect of *J*_C_ on the overall performance of the LNA, a figure of merit (*FoM*) is introduced, which can be expressed as follows:

(2) $$FoM=\frac{G_{\max}[lin]}{F_{\min}\cdot P_{\mathrm{DC}}}$$

**Figure 3:**
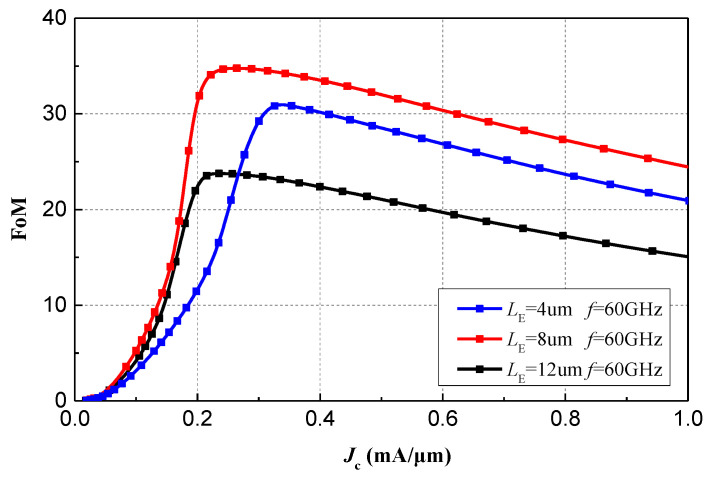
Simulated *FoM* with variations of *J*_C_ at 60 GHz and 25 °C under different *L*_E_.

**Figure 4:**
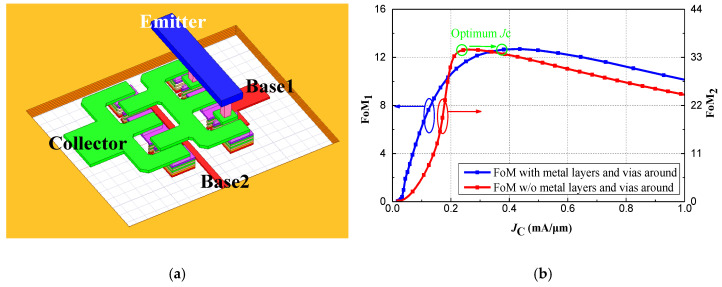
(**a**) The 3D model of first stage of LNA; (**b**) Comparation between FoM1 and FoM2 with variations of *J*_C_ at 60 GHz and 25 °C when *L*_E_ = 8 μm.

By analyzing the *J*_C_ and *FoM* values at different emitter lengths, the optimal device size and biasing state for the desired operating frequencies can be determined. As shown in Figure 3, the HBT device with an emitter length of *L*_E_ = 8 μm achieves a better *FoM* at an approximate current density of 0.24 mA/μm compared to sizes of *L*_E_ = 4 μm and *L*_E_ = 12 μm. Furthermore, in the V-band frequencies, the parasitic parameters caused by the metal interconnections and vias of the HBT devices cannot be overlooked. Therefore, it is necessary to model the passive interconnects surrounding the HBT devices and perform full-wave electromagnetic simulations. Considering the current handling capacity of a single HBT device, a parallel configuration of multiple small-sized transistors is preferred while maintaining the same emitter length. The CB-CE stage employs a parallel and staggered layout of four HBTs with an emitter length of 4 μm, which is also modeled in 3D using full-wave electromagnetic simulation software, as shown in Figure 4a. Figure 4b compares the modeled FoM1 of the interconnect lines around the HBT device with the unmodeled FoM2. It is evident that significant parasitic effects degrade the *FoM*, with the optimal current density increasing from 0.24 mA/μm to 0.38 mA/μm. Therefore, after considering the design requirements, the current density for the first phase is ultimately set at 0.32 mA/μm.

### 2.2. Interstage Bandwidth Extension

The L-shaped and Π-shaped pole tuning networks have been proven to be effective means of bandwidth extension and are widely used in broadband LNA designs [16,17]. In this design, a T-type inductive pole tuning network is employed for interstage matching to achieve bandwidth extension.

**Figure 5:**
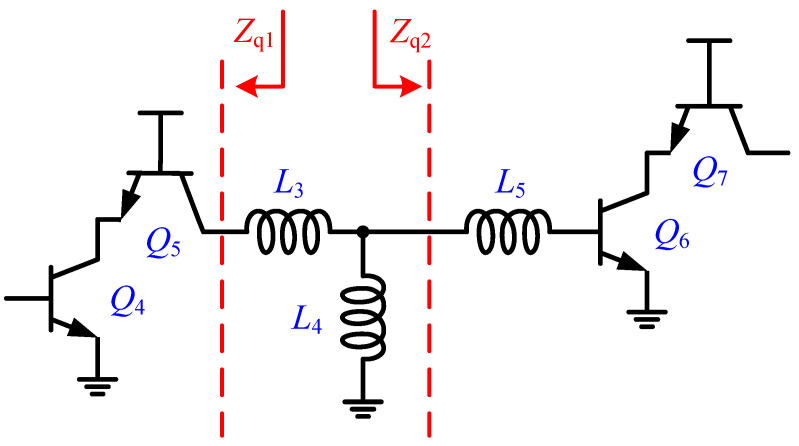
The schematic of the inter-stage T-type network.

Figure 5 presents the schematic of the inter-stage T-type pole turning network. *Z_q_*_1_ denotes the output impedance of the first stage. To illustrate the effects of *L*_3_ and *L*_4_ on the two main poles: Pole_L_ and Pole_H_, combining *L*_5_ of the T-type matching network with the input impedance of the second-stage cascode into *Z_q_*_2_. *Z_q_*_1_ and *Z_q_*_2_ can be expressed as follows:

(3) $$Z_{q1}=r_{o5}+\frac{g_{m5}r_{o4}r_{o5}+r_{o5}}{1+sC_{be5}}$$

(4) $$Z_{q2}=sL_{5}+R_{b6}+\frac{1}{sC_{be6}}+sL_{6}(C_{be6}g_{m6}+1)$$

where *r_o_*_4/5_ and *C_be_*_5/6_ are the equivalent output resistors of *Q*_4/5_ and the equivalent input parasitic capacitors of *Q*_5/6_, respectively, while *g_m_*_5/6_ is the transconductance of *Q*_5/6_. Noting that, the *Z_q_*_1_ features capacitive characteristics, while the *Z_q_*_2_ features inductive characteristics, and we define *Z_q_*_1_ = *R_q_*_1_ + 1/*sC_q_*_1_ and *Z_q_*_2_ = *R_q_*_2_ + *sL_q_*_2_ for analytical convenience. Then the simplified *Av*(*s*) of the two-stage cascode can be expressed as follows:

(5) $$Av(s)\approx \frac{-g_{m4}(sC_{q1}R_{q1}+1)[sL_{4}(R_{q2}+sL_{q2})+sL_{3}(sL_{4}+R_{q2}+sL_{q2})]}{\left(sL_{4}+sL_{q2}+R_{q2})(sC_{q1}L_{3}+C_{q1}R_{q1})+sC_{q1}L_{4}(R_{q2}+sL_{q2}\right)}$$

**Figure 6:**
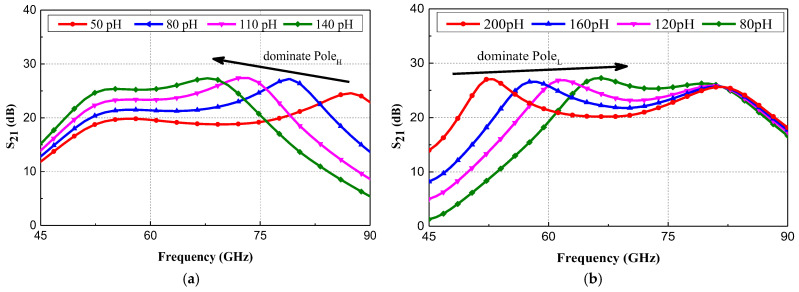
(**a**) S_21_ of the proposed LNA when *L*_3_ vary from 50 to 140 pH; (**b**) S_21_ of the proposed LNA when *L*_4_ vary from 50 to 140 pH.

The denominator of Figure 5 features two poles that are mainly dominated by *L*_3_ and *L*_4_. Figure 6 show the graphical analysis through S-parameter simulations that demonstrate the changes of the dominate Pole_L_ and Pole_H_ with the variations of *L*_3_ and *L*_4_. The increase of *L*_3_ will move Pole_H_ to lower frequency with minimal impact on Pole_L_. In contrast, the decrease of *L*_4_ will move Pole_L_ to a higher frequency with minimal impact on Pole_H_. Thus, a wideband gain–frequency response can be realized by carefully designing the parameters of the T-type inductive network to tune the positions of the high-frequency and low-frequency dominant poles.

### 2.3. Negative Temperature Coefficient Compensation Circuit

Due to the inherent factors of the silicon-based process, thermal variation will inevitably cause unexpected changes in the electron mobility and threshold voltage of the transistors, which will also affect the LNA performance. HBT devices are current-controlled devices, and the variations in collector current caused by temperature change are more pronounced. Therefore, a robust design that stabilizes gain over a wider temperature range is desirable.

**Figure 7:**
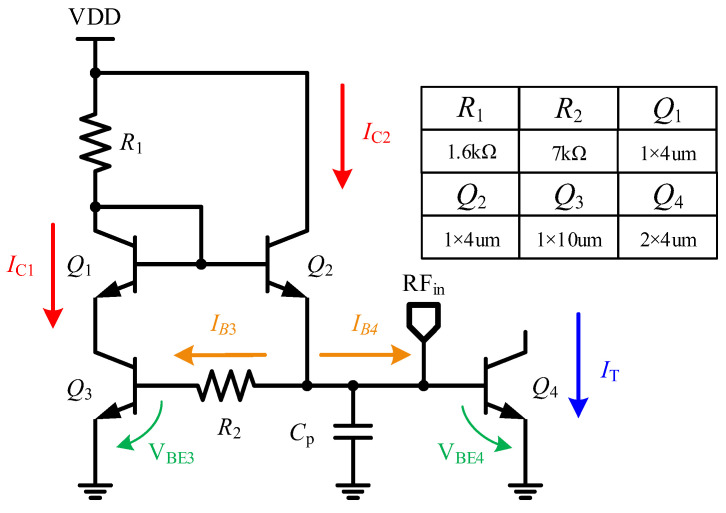
The schematic of the NTC biasing circuit in the proposed LNA.

Figure 7 is the schematic of the biasing circuit and the parameters of the HBT and resistors in the proposed LNA. The collector current of *Q*_4_ can be expressed as follows:

(6) $$I_{T}=I_{S4}\exp(\frac{V_{BE4}}{V_{T}})$$

where *V_T_* = *KT/q*, and the saturation current *I_S_*_4_ ∝ *μKTni*^2^. For convenience, *q* and *k* can be regarded as fixed coefficients, *T* is the absolute temperature, *μ* represents the carrier mobility, and *ni* denotes the intrinsic carrier concentration of silicon. Noting that *μ* ∝ *μ*_0_*T*^1+*m*^, *ni*^2^ ∝ *T*^3^exp(*−Eg/KT*) [18]. Then *I*_*S*4_ can be expressed as follows:

(7) $$I_{S4}=bT^{4+m}\exp(\frac{-E_{g}}{KT})$$

where *b* is regarded as a proportion factor; and *E*_g_ and m are assumed to be constant and the values of these in HBT are approximately 1 eV and −1.5, respectively. Substituting (7) into (6) we obtain the following:

(8) $$I_{T}=bT^{4+m}\exp(\frac{-E_{g}+qV_{BE4}}{KT})$$

**Figure 8:**
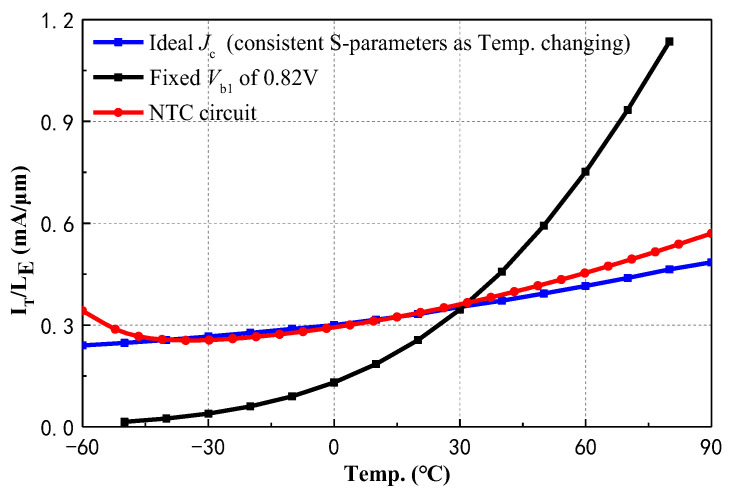
Comparison of *J*_C_ in basing situations of fixed Vb1, NTC circuit, and ideal *J*_C_.

From (7) and (8), high sensitivity of *I_S_*_4_ to temperature greatly affects robustness of HBT to temperature. It is essential to introduce a compensation biasing circuit with a negative temperature coefficient (NTC) to ensure *Q*_4_ operates at an optimal *J*_C_ as the temperature changes and, referring to the method in [14], the optimal *J*_C_ at each temperature should be obtained first. As shown in Figure 8, the blue curve represents the ideal *J*_C_ that obtained through simulations to ensure the same S-parameter at each temperature. The black curve illustrates the *J*_C_ with temperature without the temperature compensation circuit. Although the optimal *J*_C_ is achieved at 25 °C, there are significant variations in *J*_C_ with temperature fluctuations, leading to considerable deviations from the ideal curve. The red curve represents the *J*_C_ after implementing the NTC circuit. While there are slight deviations from the ideal curve at −60 °C and 90 °C, the fitting is ideal within the range of −55 to 85 °C, which indicates that the S-parameters will not experience significant variations.

In Figure 7, *R*_2_ is a resistor that exhibits good temperature characteristics, and *C*_p_ is bypass capacitance to minimize the influence of the biasing circuit. *R*_1_, *Q*_1_, and *Q*_2_ form a conventional current mirror structure. So, assuming *I_E_*_2_ = *nI_E_*_1_ and *n* is a constant that shows slight sensitive to temperature. *Q*_4_ relies on the base current *I*_B4_ to control the collector current *I*_T_, with a current gain of *β*_4_. Therefore, Equation (6) can be derived.

(9) $$I_{T}=\beta_{4}(n-\frac{1}{\beta_{3}})I_{E1}$$

The KVL equation between *Q*_3_ and *Q*_4_ can be derived as follows:

(10) $$V_{BE4}-V_{BE3}=R_{2}\frac{I_{E1}}{\beta_{3}}$$

*I*_T_ can be derived by substituting (6) and (9) into (10), as follows:

(11) $$I_{T}=\beta_{4}(n-\frac{1}{\beta_{3}})\frac{V_{T}\beta_{3}}{R_{2}}\ln^{\frac{I_{E4}I_{S3}}{I_{E3}I_{S4}}}$$

The temperature-sensitive parameters, *I_E_* and *I_S_*, cancel the effect of *T* in *I_E_*_4_*I_S_*_3_/*I_E_*_3_*I_S_*_4_. Additionally, the slow variation of logarithmic ln(*I_E_*_4_*I_S_*_3_/*I_E_*_3_*I_S_*_4_) with *T* also reduces the impact of the temperature.

**Figure 9:**
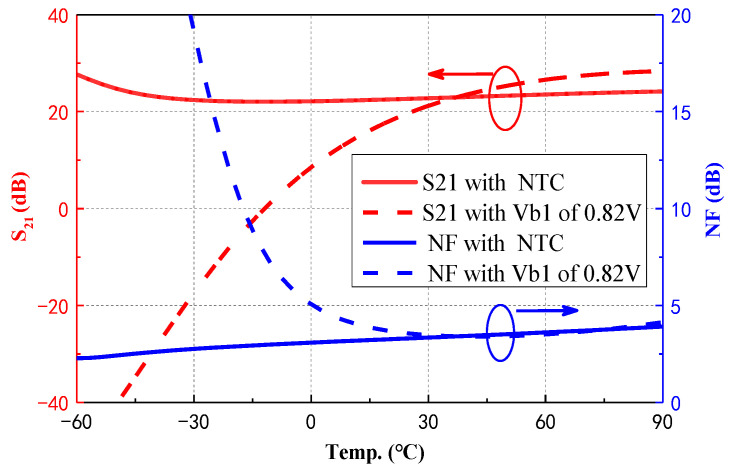
Comparison between simulated S_21_ and NF in basing situations of fixed Vb1 and NTC at 55 GHz.

To intuitively demonstrate the compensatory effects of the proposed NTC on the LNA performance, Figure 9 presents simulated results of small-signal gain (S_21_) and NF as a function of temperature at a fixed bias voltage under NTC conditions at an operating frequency of 55 GHz. In the uncompensated scenario, the gain and noise figure of the LNA sharply degrade as the temperature drops to around −15 °C, primarily due to the pronounced temperature sensitivity of the HBT devices. In contrast, with the implementation of the proposed NTC, both gain and NF remain stable over a broad temperature range from −60 °C to 90 °C, demonstrating remarkable robustness.

## 3. Experimental Validation and Results

**Figure 10:**
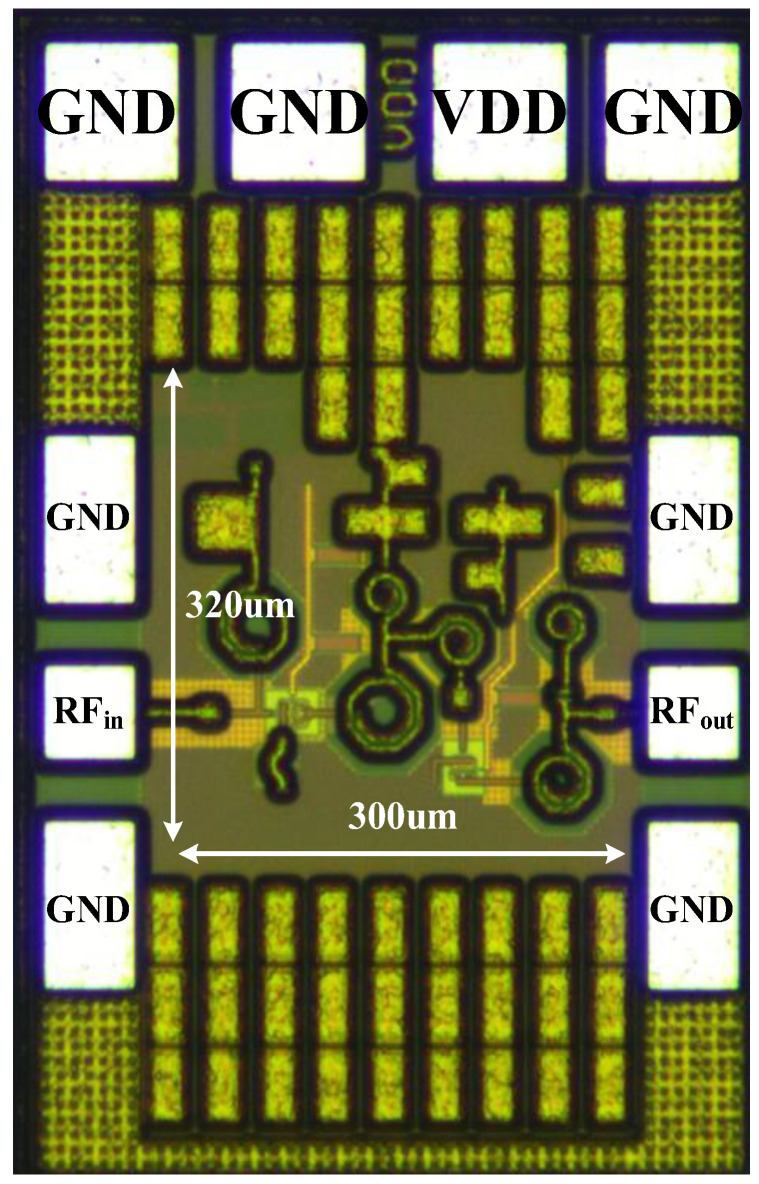
Microphotograph of the LNA chip.

The proposed temperature compensation LNA is fabricated using a 0.13 μm SiGe BiCMOS process. Figure 10 shows the die micrograph of the proposed LNA. The whole chip occupies a silicon area of 0.49 mm × 0.76 mm including all testing pads and a core area of 0.3 mm × 0.32 mm.

An MPI TS200-SE probe station equipped with ground–signal–ground (G-S-G) probes with a 150 µm pitch was used for on-wafer testing and the calibration was performed using the standard short-open-load-through (SOLT) method. The S-parameters of LNA were measured using a Keysight PNA-X N5247B network analyzer(Keysight Technologies, Inc., Santa Rosa, CA, USA) with a maximum frequency range of 10 MHz to 67 GHz. Due to the limited calibration accuracy of the PNA-X N5247B network analyzer near its upper frequency limit of 67 GHz, the measurements in this work were conducted between the range of 40 GHz and 65 GHz.

**Figure 11:**
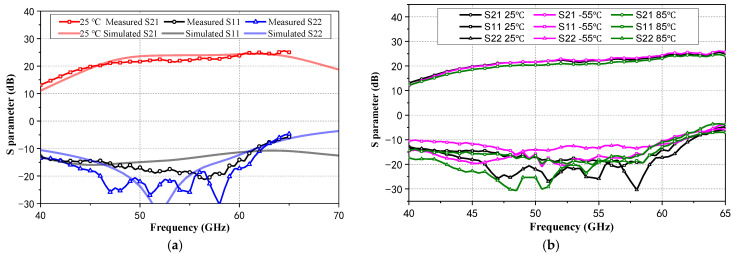
(**a**) Comparison between simulated and measured S-parameters at 25 °C; (**b**) Measured S-parameters from the proposed LNA at −55 °C, 25 °C, and 85 °C.

Figure 11a presents the comparison between simulated and measured S-parameters at 25 °C, which exhibits similar trends. From measured results, the LNA achieves a peak gain of 25 dB, a 3 dB bandwidth exceeding 11 GHz, and both S_11_ and S_22_ are better than −10 dB within 40~61 GHz. The measured S-parameters of the proposed LNA at −55 °C, 25 °C, and 85 °C are demonstrated in Figure 11b. Owing to the NTC basing network, the LNA’s measured S-parameters exhibit excellent robustness to temperature, with ΔGain less than 1.5 dB over 40~65 GHz.

**Figure 12:**
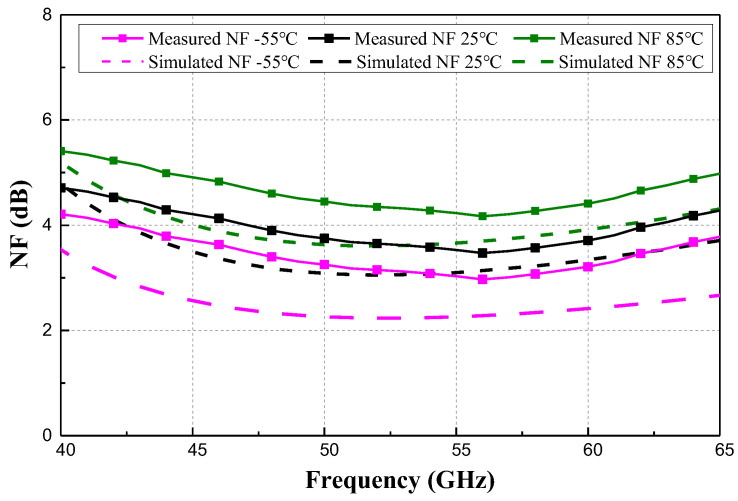
Simulated and measured NF at −55 °C, 25 °C, and 85 °C.

Figure 12 shows the measured and simulated NF of the proposed LNA at −55 °C, 25 °C, and 85 °C. At 25 °C and 85 °C, the measured results match well with the simulation. However, at −55 °C, the measured NF exhibits a deviation of 0.7 dB from the simulation. One factor for the discrepancy could be the decreasing of measured S_21_ at −55 °C compared to the simulation one, which leads to inadequate noise suppression.

**Figure 13:**
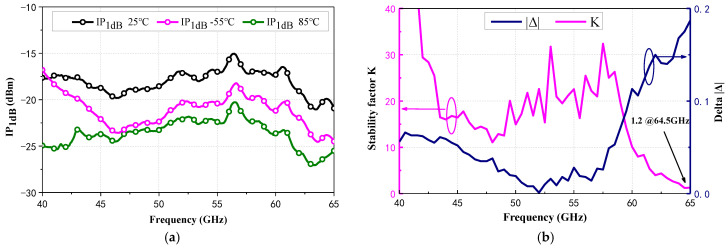
(**a**) Measured IP_1dB_ at −55 °C, 25 °C, and 85 °C; (**b**) Measured stability factor K and delta |△|.

Figure 13a displays the measured input 1 dB compression point (IP_1dB_), which indicates the amplifier’s linearity. Unlike the measured S-parameters, the IP_1dB_ is optimal at 25 °C. This could be attributed to two factors: (1) A high gain at −55 °C will sacrifice linearity. (2) The use of an NTC basing network, which adjusts *J*_C_ to ensure good consistency in the S-parameters across −55~85 °C, may not be optimal for linearity. Within the measured frequency range of 40 to 65 GHz, the stability factor *Kf* was consistently greater than 1.2, while the delta Δ remained under 1. These results demonstrate that the proposed wideband LNA achieved stability in all operating conditions.

Table 1 summarizes the performance of the proposed LNA and compares it with other recently reported LNAs. The temperature compensation circuits proposed in references [12,14] effectively improve the LNA performance, but their designs are relatively complex and occupy a large area, while also significantly degrading other LNA parameters. The 60 GHz LNA in reference [12] experiences a 2.1 dB degradation in NF at high temperatures. The size of the temperature compensation circuit in reference [14] is 0.168 mm^2^, accounting for one-third of the total chip area. The LNA reported in [19] experienced a 5 dB gain degradation at temperatures ranging from −5 to 85 °C, indicating significant room for improvement. The proposed LNA exhibits good stability in key performance metrics, such as gain, NF, and bandwidth, when facing variations in environmental temperature, with a compact core area of only 0.09 mm^2^. Compared to LNAs without temperature compensation, such as [9,10,20], the proposed LNA shows superior gain and bandwidth at room temperature. Furthermore, it maintains a high gain of over 20 dB with a less than 1.5 dB variation across a temperature range of −55 to 85 °C. Due to system design specifications requiring a 3.3 V operating voltage, the proposed LNA exhibits relatively high-power consumption.

## 4. Conclusions

An adaptive temperature compensation technology has been successfully implemented in a compact V-band wideband LNA fabricated using a 130 nm SiGe BiCMOS process. This innovation enables the LNA to exhibit excellent temperature robustness over a broad range from −55 °C to 85 °C. The V-band LNA demonstrates a gain variation of less than 1.5 dB, an NF variation of less than 1.2 dB, and an IP_1dB_ variation of less than 5 dBm over measured frequencies from 40 GHz to 65 GHz. The measured DC power consumptions at −55 °C, 25 °C, and 85 °C are 22.3 mW, 27.6 mW, and 34.4 mW, respectively, with a supply voltage of 3.3 V. At room temperature, the LNA demonstrates a gain of 25 dB, an NF of 3.5 dB, and an IP_1dB_ of −17.5 dBm. The LNA chip has a total silicon area of 0.37 mm^2^, including all test pads, with the core area being only 0.09 mm^2^.

## Figures and Tables

**Table 1:** Performance summary and comparison with reported V-band LNAs and temperature compensation amplifier.

Ref.	Process	Temp. (°C)	Peak Gain (dB)	ΔGain (dB)	Temp. Coefficient ΔGain_max_/S_21 RT_/ΔT (dB/°C) [12]	BW_3dB_ (GHz)	BW_5dB_ (GHz)	IP_1dB_ (dBm)	NF_min_ (dB)	*P*_DC_ (mW)	Core Area (mm^2^)
[12]	65 nm CMOS	−20	10.4	<1.7	1.4 × 10^−3^	>11 (56~67) *	>13.5 (53.5~67) *	\	\	\	0.72 ^&^
		25	9.9			>11.5 (55.5~67)	>13.5 (53.5~67)*	\	3.9	17.2	
		100	8.8			>11.5 (55.5~67)	>13.5 (53.5~67) *	\	6.0	23.4	
[14]	90 nm CMOS	−45	23.6	<1.2	0.29 × 10^−3^	N/A	N/A	\	\	\	0.5 ^&^
		25	23.8			4 (24.5~28.5)	5 (24~29) *	\	\	25.2	
		125	23			N/A	N/A	\	\	\	
[19]	90 nm CMOS	−5	22.5	<5	2.6 × 10^−3^	10 (55~65) *	>11 (54~65) *	\	\	\	1.06 ^&^
		25	21			8 (55~63) *	>11 (54~65) *	−20 *	6.5	49	
		85	17.5			11 (54~65) *	>12.5 (52.5~65) *	\	\	\	
[9]	45 nm RF-SOI	25	20.4	\	\	13.6 (54.4~68)	18 (53~71)	−23.6	5	8.6	0.138
[10]	40 nm CMOS	25	19.8	\	\	10 (55~65)	13 (54~67)	−29.5	6	18	0.22 ^&^
[20]	65 nm CMOS	25	12.8	\	\	10 (55~65)	>12 (54~66)	\	3.6	8.8	0.33
**This work**	**130** **nm** **BiCMOS**	**−** **55**	**25.5**	**<1.5**	**0.4 × 10** **^−^** **^3^**	**>13** **(52~65)**	**>18.5** **(46.5~65)**	**−** **20**	**3.0**	**22.3**	**0.09**
		**25**	**25**			**>11** **(54~65)**	**>19.5** **(45.5~65)**	**−** **17.5**	**3.5**	**27.6**	
		**85**	**24.4**			**>10** **(55~65)**	**>18** **(47~65)**	**−** **22.5**	**4.2**	**34.4**	

* Estimated values from papers; ^&^ DC pads are included.

## Data Availability

The data presented in this work are available within the article.
